# A conditional GAN-based approach for enhancing transfer learning performance in few-shot HCR tasks

**DOI:** 10.1038/s41598-022-20654-1

**Published:** 2022-09-29

**Authors:** Nagwa Elaraby, Sherif Barakat, Amira Rezk

**Affiliations:** Information Systems Department, Faculty of Computers and Information, Mansoura University, Mansoura, 35516 Egypt

**Keywords:** Computer science, Information technology

## Abstract

Supervised learning with the restriction of a few existing training samples is called Few-Shot Learning. FSL is a subarea that puts deep learning performance in a gap, as building robust deep networks requires big training data. Using transfer learning in FSL tasks is an acceptable way to avoid the challenge of building new deep models from scratch. Transfer learning methodology considers borrowing the architecture and parameters of a previously trained model on a large-scale dataset and fine-tuning it for low-data target tasks. But practically, fine-tuning pretrained models in target FSL tasks suffers from overfitting. The few existing samples are not enough to correctly adjust the pretrained model’s parameters to provide the best fit for the target task. In this study, we consider mitigating the overfitting problem when applying transfer learning in few-shot Handwritten Character Recognition (HCR) tasks. A data augmentation approach based on Conditional Generative Adversarial Networks is introduced. CGAN is a generative model that can create artificial instances that appear more real and indistinguishable from the original samples. CGAN helps generate extra samples that hold the possible variations of human handwriting instead of applying traditional image transformations. These transformations are low-level, data-independent operations, and only produce augmented samples with limited diversity. The introduced approach was evaluated in fine-tuning the three pretrained models: AlexNet, VGG-16, and GoogleNet. The results show that the samples generated by CGAN can enhance transfer learning performance in few-shot HCR tasks. This is by achieving model fine-tuning with fewer epochs and by increasing the model’s $F1-score$ and decreasing the Generalization Error $(E_{test})$.

## Introduction

Handwritten Character Recognition (HCR) is one of the foremost vital fields in the computer vision domain. It is concerned with building machine learning models that can best recognize and distinguish written characters by humans. The necessity for such models has increased in most banking, postal, medical, and teaching services. Resorting to deep learning by most researchers in building HCR models is an optimal direction because of its unprecedented ability to achieve performance near human-level performances^1–4^. However, deep learning is valid and provides efficient spatial understanding and deep features only in the presence of sufficient training samples (thousands or tens of thousands per class)^5^.

Consequently, building deep learning models for Few-Shot Learning (FSL) in the HCR has become a challenge for researchers. The precise meaning of FSL is the cases in which only a few training samples are available to build a model^6,7^. It is considered a simulation for the human brain’s ability to learn new object categories from a few instances. Humans can distinguish written characters, text, and languages by viewing a few examples of them or just a first look. FSL is useful for building more generalized models with fewer costs, but practically its tasks are nontrivial. The existing few samples in FSL tasks are considered support sets and not training sets and will not be sufficient to build a deep network from scratch.

**Figure 1:**
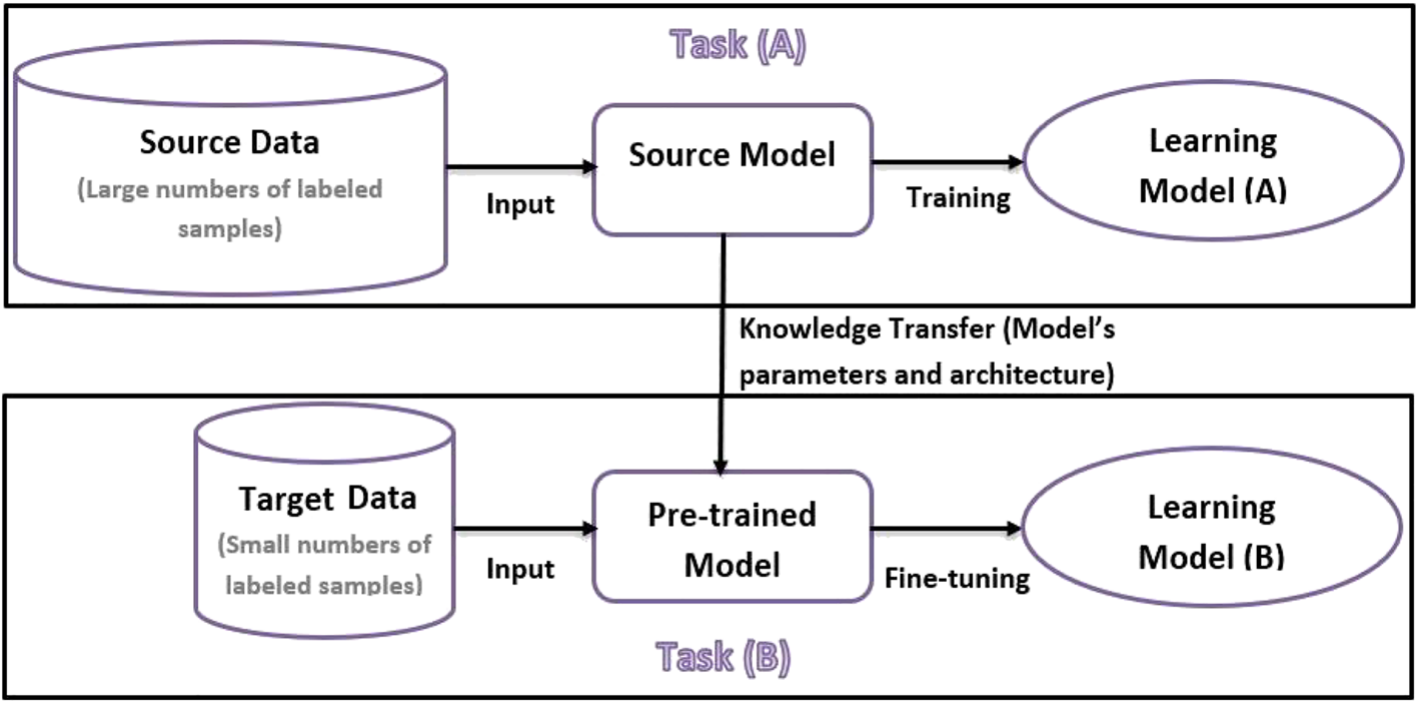
Transfer learning methodology creates high-performing learners by extracting knowledge learned from previous tasks and applying it to new related low-data tasks.

Applying transfer learning to dispense with the difficulty of building new deep networks for few-shot HCR tasks is recommended^8–10^. As shown in Fig. 1, transfer learning follows the principle of “instead of building a deep network from scratch for a low-data target task, borrow the architecture and parameters of a previously pretrained network on a related source task.” This methodology allows the reuse of a previously trained model on sufficient training data and fine-tuning it for FSL tasks. Fine-tuning attends to convert the pretrained model to a model that best fits the new task. Achieving the best fitting in an FSL task has become a challenge since fine-tuning with few training samples usually causes model overfitting.

Overfitting (also called Generalization Error) means that the model loses its generalization ability from training data to unseen data^11,12^. The core reason for this phenomenon is the quantity and quality of the training samples. Fine-tuning the model parameters with a few training samples make it a less representative view of the FSL target task. Enlarging the size of the training data by applying a data augmentation technique is an effective solution to the overfitting problem^13^. Data augmentation has succeeded in saving the costs of collecting labeled data and overriding data neediness without any human intervention^14^. Traditional dataset expansion methods are the most commonly applied methods for data augmentation^15–18^.

Traditional dataset expansion methods create slightly edited copies of the existing training data by applying one or more traditional transformations, such as rotation, translation, flipping, sharpening, and color changing. These methods are simple, easy to implement, and save disk space in real-time implementation^19^. But applying traditional data augmentation methods in HCR tasks has two primary drawbacks:

1. The traditional transformations are low-level, data-independent operations and can only produce augmented samples with limited diversity^20^. The data variations presented using these methods may not cover actual variations in character handwriting. The writing form of characters varies among humans, and sometimes the writing style of the same individual differs from time to time. The representation of these variations is impossible by just a traditional transformation.
2. There are no unified transformations as a data augmentation model for all HCR tasks^16,21^. It is a task-dependent problem. Therefore, it is necessary to conduct several trial-and-error experiments for each HCR task to determine which transformations are suitable for increasing its performance.

Such drawbacks motivate the need to generate synthetic samples that can hold possible variations in human handwriting and, concurrently, appear more realistic and indistinguishable from the original samples. Using Generative Adversarial Networks (GANs) may achieve the proposed motivation. GAN consists of two networks trained simultaneously: generator and discriminator^22^. The generator adds random noise to the input to produce synthetic samples with the same structure and distribution as real samples. Then, the generated and real samples are forwarded to a discriminator that works as a classification network. If the discriminator succeeds in distinguishing between the two types of samples, the generator loss is considered to update the generator network. Updating the generator helps in making it produce synthetic samples that appear more real and fall at the discriminator fault point.

General GAN works in an unsupervised mode. It can generate random images from the domain without control over which data categories should be generated^23^. Conditional GAN (CGAN) is the supervised version of GAN. In CGAN, the generator and discriminator are trained under a condition that is usually a class label^24^. If GAN performs image generation, then we can say that CGAN achieves a targeted image generation. Consequently, it is considered an improvement for the general GAN. It helps in preserving stable and faster training and generating better-quality artificial data.

In this study, we introduce a data augmentation approach based on CGAN to solve the overfitting problem, which occurs when applying transfer learning in few-shot HCR tasks. First, CGAN is trained to generate synthetic samples for each character’s class to provide additional samples with the possible variations of human handwriting. Then, the generated samples by CGAN are added to the existing few ones before fine-tuning the transfer learning model. Thus, the model during fine-tuning has sufficient variations for the input that helps adjust its parameters correctly and acquire the generalization ability for new-unseen test samples.

The remainder part of the study is structured into sections as follows. A literature review is presented in “Literature review” section. “Basic concepts” section presents the basic concepts of generative and discriminative models, GANs, CGANs, and transfer learning. “Using CGAN in fine-tuning transfer learning models for few-Shot HCR tasks” section introduces the proposed framework for using CGAN in fine-tuning transfer learning models for few-shot HCR tasks. “Experimental results and discussion” section consists of the experimental results and the discussion. The conclusions and suggestions for future study are presented in “Conclusion and future work” section.

## Literature review

FSL is a subarea that puts deep learning performance in a gap. Building a deep network from scratch and adjusting its hyperparameters, such as bias and weights, entails numerous labeled training samples. Expanding the superior performance of deep models to include FSL tasks can be performed using transfer learning^6,25,26^. Chen et al.^27^ introduced a transfer learning idea for enhancing the accuracy of Electrocardiogram (ECG) classification with small datasets. The effectiveness of this idea was evaluated using First China’s ECG Intelligent Competition dataset. Han and Jin^28^ showed that the accuracy and robustness of small-sample image recognition could be improved using the hybrid training mode of Convolutional Neural Networks (CNNs) and transfer learning. Alzubaidi et al.^29^ proposed a novel transfer learning approach to fill the performance gap of deep learning models when there was a lack of training data in medical imaging tasks. Jing et al.^30^ suggested a feature transfer framework that depends on transferring knowledge from related fields to facilitate and reduce the challenges of fault diagnosis with small samples.

However, practically, applying transfer learning in few-shot HCR tasks suffers from overfitting. Human handwriting is inconsistent. The writing styles of humans vary according to the circumstances. Fine-tuning any transfer learning model with few shots of handwritten samples allows it to achieve a high generalization error in recognizing unseen test samples. Early stopping, regularization, and data augmentation are three state-of-the-art strategies for solving the overfitting problem^11,31^ . The early stopping strategy states that training must be stopped before the performance decreases^32,33^. The regularization strategy concludes that the network must preserve only neurons that hold useful features^34,35^. Finally, the data augmentation strategy guarantees the network’s performance by adjusting its hyperparameters sets with a large amount of data^16^.

In our study, we recommend a data augmentation strategy to avoid the overfitting problem that occurs when fine-tuning pretrained deep networks for few-shot HCR tasks. We consider the core reason for the overfitting problem in this study to be the disability of correctly adjusting network parameters in the presence of a few training samples. Augmenting data may be developed based on basic image manipulations or generative modeling.

### Data augmentation based on basic image manipulation

This type of augmentation deliberates by applying traditional image transformations to the available training samples to generate slightly edited copies of them. Traditional transformations are classified into geometric and photometric transformations^36^. Geometric transformations are interested in changing the image geometry by moving its pixel positions. Rotation, translation, and flipping are examples of geometric transformations. However, photometric transformations are concerned with altering the image’s color properties by shifting each pixel value to a new one. Similarly, color jittering, contrast changing, and edge enhancement are examples of photometric transformations.

Zhang et al.^37^ solved the FSL problem in ear recognition using the traditional data augmentation methods. They augmented the number of training samples up to a factor of 100 by applying horizontal flipping, cropping, scaling, rotating, and contrast-changing transformations. Experiments proved that the proposed solution created a flexible model that could adapt to new test data and perform fast recognition. However, it was not tested in open-set ear recognition problems, which are highly challenging. Noon et al.^38^ explored the effect of traditional data augmentation methods in avoiding the overfitting problem when fine-tuning a pretrained DenseNet-121 model for plant leaf disease recognition. They performed the experiments using the combinations of several rotations, width shift, height shift, zoom, horizontal flip, and vertical flip transformations. The results showed that the network generalization was best for the combination of width shift and height shift transformations. However, the combination of zoom and rotation transformations makes the network highly prone to overfitting. Joseph and George^19^ compared the performance of traditional data augmentation methods with two execution modes. The main idea of the comparison was to determine which mode was best for tackling the problem of training data scarcity in the HCR. The first mode was offline augmentation, in which traditional transformations were applied to the existing training examples before training and saved in the disk for use during the training. The second mode was real-time augmentation, in which traditional transformations were applied during training without saving to the disk. Experiments showed that the real-time mode helps CNNs achieve better accuracy by exceeding low resources compared with the offline mode. However, the applied transformations in each mode are different, which makes the comparison unfair. Ahmad et al.^39^ suggested applying traditional data augmentation methods to classify novel COVID-19 when sufficient chest X-ray images are absent. The applied transformations include random rotation, random horizontal reflection, random vertical reflection, random horizontal shear, and random vertical shear. They used generated augmented data for hyperparameter tuning in several transfer learning models. The results showed that the applied transformations helped increase the performance significantly. However, augmented X-ray images are still not highly accurate as benchmarks for identifying COVID-19 infections in patients. Fabian et al.^40^ introduced a data augmentation pipeline to reduce the costs of collecting training data for accelerated Magnetic Resonance Imaging (MRI). The proposed pipeline was a combination of pixel preserving and general affine transformations and applied to different small-sample datasets. Then, they used the augmented datasets to train an end-to-end VarNet model. The results confirmed that applying traditional data augmentation in the low-data regime is an optimal surrogate for generating flexible models against overfitting. However, the challenge of this study was how to find the optimal augmentation strength throughout training. De la Rosa et al.^41^ studied the effect of data augmentation on the performance of a ResNet-50 model in defect classification problems. Especially, in cases where the volumes of training images and balanced classes are small. They applied scaling, rotation, translation, and flipping transformations to increase the volume of defect images. Similarly, they performed seven experiments to evaluate the model performance as they increased the dataset to twice as many images as the previous experiment. The results showed that the F1 score of the model increased every time the dataset volume increased with augmented images. However, this study does not regard the Generalization Error for performance evaluations.

### Data augmentation based on generative modeling

This type of augmentation depends on generating synthetic data that hold characteristics to the original data. Generative models can create artificial instances that appear more real and indistinguishable from the original data. Antoniou et al.^42^ developed a Data Augmentation GAN (DAGAN) model to generate reliable synthetic data for the low-data regime tasks. They used transfer learning to build the structure of the proposed DAGAN. A combination of two standard networks, UNet, and ResNet, builds the generator, whereas the DensNet architecture builds the discriminator. They used the DAGAN to generate artificial samples for the human faces and handwriting domains and used the generated samples to train a standard Stochastic Gradient Descent Neural Network (SGDNN). The results showed that the proposed DAGAN significantly improved the classification accuracy in each domain. Further evaluations of the developed GAN in FSL are necessary. Frid-Adar et al.^43^ proposed an augmentation approach to solving the problem of overfitting in training deep networks for low-data medical recognition problems. The proposed approach consists of traditional data augmentation methods and GAN. First, they used the traditional methods to increase the training data in terms of size and diversity. Then, GAN was applied to generate synthetic data augmentation. The results showed that using GAN with the traditional methods increased the performance of CNN to $7\%$ compared with using only traditional methods. However, using GAN to generate artificial images for each class is a time-consuming task. Mondal et al.^44^ studied the perspective of FSL in segmenting 3D multimodal medical images. They analyzed two different GAN architectures to determine which one was appropriate to significantly improve the segmentation performance. The first architecture was the Feature Matching GAN (FM GAN), which used the feature matching loss for training the generator. The second one was the Bad-GAN, where unlabeled and artificial images were not separated in the generator. The empirical results showed that FM GAN outperformed Bad-GAN in segmenting 3D multimodal brain MRI images. However, further experiments are required for the FM Bad-GAN, as Bad-GAN is essential for good semi-supervised learning. Guan and Loew^45^ proposed a solution of two deep-learning-based technologies for developing breast cancer detection systems when training examples are small. The first technology focused on training a GAN network to generate synthetic mammographic images. The second focused on applying transfer learning using the pretrained VGG-16 model. The experiments showed that combining these two steps helps to obtain the best classification performance. GAN avoided overfitting in the pretrained network, and transfer learning increased the speed of training approximately 10 times faster than training CNN from scratch. However, by replacing GAN with its supervised version, CGAN may reduce the time needed to generate targeted images for each class. Zhang et al.^46^ proposed a Deep Adversarial Data Augmentation (DADA) technique to solve the overfitting problem in the ill-posed extremely low-data regimes. The technique was built by training a supervised GAN and applying the 2K loss to the GAN’s discriminator. The experiments showed the power of the GAN in generating new training data and enforcing fine-grained classification. However, in evaluations, the proposed DADA has yet to be applied to real-world tasks, such as military, satellite, and biomedical image classification. Jha and Cecotti^18^ suggested using generative networks to avoid generalization errors when training a network with small labeled examples in handwritten digit recognition tasks. Therefore, GAN was used to generate new artificial images for every single class in each task. The results showed that the suggested augmentation approach caused a substantial gain in accuracy. However, they noticed that the overall performance might decrease when they added too many artificial images to the original training examples. Yunusa et al.^47^ introduced a generative augmentation framework to increase the CNN’s ability to recognize rice leaf diseases when large quality datasets are absent. They built a StyleGAN2 Adaptive Discriminator Augmentation (SG2-ADA) architecture to be an improvement to the vanilla GAN by regularizing the generator, redesigning the generator normalization, and modifying the progressive growing. They used the SG2ADA to generate synthetic rice leaf disease images for training Faster Region-Based CNN (Faster- RCNN) and Single Shot Detector(SSD) models. The observations from the experimental results told that the SG2-ADA produces better-quality artificial images and leads to good recognition when compared with the traditional augmentation methods. However, experiments miss comparing the performance of StyleGAN2 and the vanilla GAN architecture. Asghar et al.^48^ studied the overfitting problem that occurred when CNNs used to detect COVID-19 cases in the scarcity of X-ray images. They solved this problem by exploring two data augmentation approaches, the first was the traditional transformations and the second was the GAN. They evaluated the generated samples by the two approaches in training InceptionV3, Resnet101, DenseNet-121, Xception, and QuNet models. The experimental results showed that the highest detection accuracy is achieved by Xception and QuNet models when applying the traditional transformations and by the QuNet model when using GAN. However, the experimental observations couldn’t conclude the perfect augmentation approach to detect the novel COVID-19.

Table 1 summarizes the above mentioned studies. Although, these studies applied data augmentation strategies either depend on basic image manipulations or generative modeling. Thus, this study introduced a data augmentation approach based on generative modeling to mitigate the overfitting problem when applying transfer learning in few-shot HCR tasks. Preferring generative modeling to basic image manipulations generates synthetic samples that hold the possible variations in handwriting. A flooding network with sufficient data variations during fine-tuning helps acquire the generalization ability. Most of the recent studies that applied generative modeling used GANs. Training GAN in supervised tasks like HCR is time-consuming. GAN trained on each class separately to generate samples belonging to that class. Therefore, CGAN is the proposed generative augmentation approach in this study. Replacing GAN with its supervised version avoids its limitations and generates better-quality artificial samples with stable and faster training.

**Table 1:** A comparison of some recent studies concentrating on applying data augmentation to avoid the overfitting problem in low-data regimes.

Reference	Augmentation Strategy	Classification Model	Task	Advantages	Limitations
Antoniou et al.^42^	DAGAN	Standard SGDNN	Low-data regime tasks	The proposed DAGAN significantly improves the classification accuracy in the human faces and handwriting domains	Further evaluations for the developed GAN architecture need to be made in FSL
Frid-Adar et al.^43^	Traditional transformations and GAN	CNN model	Low-data medical recognition tasks	Using GAN with the traditional methods increases the performance of CNN to 7% compared with using only traditional methods	Using GAN to generate artificial images for each class is a time-consuming task
Mondal et al.^44^	FM GAN and Bad-GAN	CNN model	FSL in segmenting 3D multimodal medical images	FM GAN outperforms the performance of classical GAN and Bad GAN	Further experiments are required for the FM-Bad GAN, as Bad-GAN is essential for good semi-supervised learning
Zhang et al.^37^	Horizontal flipping, cropping ,scaling, rotating, and contrast changing transformations	Different architectures of CNNs	FSL in ear recognition	The applied transformations create flexible CNN that can adapt to new test data and perform fast recognition	The applied transformations are not tested in open-set ear recognition problems which are highly challenging
Guan and Loew^45^	GAN	VGG-16 model	Breast cancer detection systems when training examples are small	GAN avoids overfitting in the pretrained network and transfer learning increases the speed of training approximately 10 times faster than training CNN from scratch	Using GAN for generating artificial images for each class is a time-consuming task
Noon et al.^38^	Rotation, width shift, height shift, zoom, horizontal flip, and vertical flip transformations	DenseNet-121 model	Plant leaf disease recognition with small datasets	The network generalization is best for the combination of width shift and height shift transformations	The combination of zoom and rotation transformations makes the network highly prone to overfitting
Joseph and George^19^	Offline and real-time traditional transformations	CNN model	HCR with data scarcity	The real-time augmentation helps CNNs achieve better accuracy by exceeding low resources compared with the offline augmentation	The applied transformations in each mode are different, this made the comparison not fair
Zhang et al.^46^	DADA	CNN model	Ill-posed extremely low-data regimes	Applying the 2K loss to GAN’s discriminator boosts the performance	The proposed DADA has yet to be applied to real-world tasks, such as military, satellite, and biomedical image classification.
Jha and Cecotti^18^	GAN	CNN model	Handwritten digit recognition tasks with low number of labeled samples	The recommended augmentation approach causes a substantial gain in accuracy	The overall performance may decrease when too many artificial images added to the original training examples
Ahmad et al.^39^	Random rotation, random horizontal reflection, random vertical reflection, random horizontal shear, and random vertical shear transformations	MobileNet, ResNet50, and InceptionV3 models	Classifying novel COVID-19 when sufficient chest X-ray images are absent	The applied transformations help increase the performance significantly	The augmented X-ray images are still not highly accurate as benchmarks for identifyingCOVID-19 infections in patients
Fabian et al.^40^	A combination of pixel and general affine preserving transformations	End-to-end VarNet model	Accelerated MRI reconstruction on small datasets	The proposed data augmentation pipeline improves the model robustness against various shifts in the test distribution	This study faces a challenge which is how to find the optimal augmentation strength throughout training
De la Rosa et al.^41^	Scaling, rotation, translation, and flipping.	ResNet-50 model	Small sample defect classification problems	The F1 score of the model increases every time the dataset volume increased with augmented images	This study does not regard the Generalization Error for performance evaluations
Yunusa et al.^47^	SG2-ADA	Faster- RCNN , and SSD models	Rice leaf diseases when large quality datasets are absent	The SG2-ADA produces better-quality artificial images and leads to good recognition	Experiments miss comparing the performance of StyleGAN2 and the vanilla GAN architecture
Asghar et al.^48^	Zoom, horizontal shift, vertical shift, and rotation transformation, and GAN	InceptionV3, Resnet101, DenseNet-121, Xception , and QuNet	Data scarcity problem in detecting COVID-19 cases	The highest detection accuracy is achieved byXception and QuNet models when applying the traditional transformations and by QuNet when using GAN	The experimental observations couldn’t conclude the perfect augmentation approach to detect the novel COVID-19

## Basic concepts

### Generative and discriminative models

Both the generative and discriminative models are probabilistic. Generative models apply the joint probability distribution using Eq. (1) to learn the input data distribution^49^. Learning data distribution enables the model to extract potential features and determine the process of generating the data. Therefore, generative models provide new artificial data with the same distribution as real data. The generated synthetic data obtained using the generative models are plausible and different from real data of the domain.

1 $$\begin{aligned} p(x,y) = p(x) \times p(y) \end{aligned}$$

**Figure 2:**
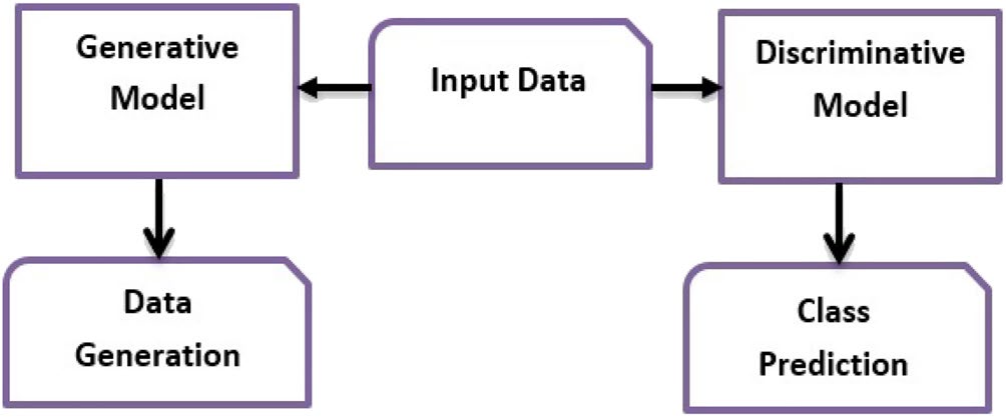
Difference between generative and discriminative modeling in dealing with input data.

Alternatively, discriminative models apply the conditional probability distribution according to Eq. (2) to learn how to map inputs (*x*) to their class labels (*y*)^49^. The formatted citation’s main goal of discriminative models is to find decision boundaries between classes. Thus, it can determine the class of new unknown data and detect outliers, but cannot generate data. Figure 2 reveals the difference between generative and discriminative models in dealing with the input data.

2 $$\begin{aligned} p(x|y) = \frac{p(x,y)}{p(y)} \end{aligned}$$

### GANs

**Figure 3:**
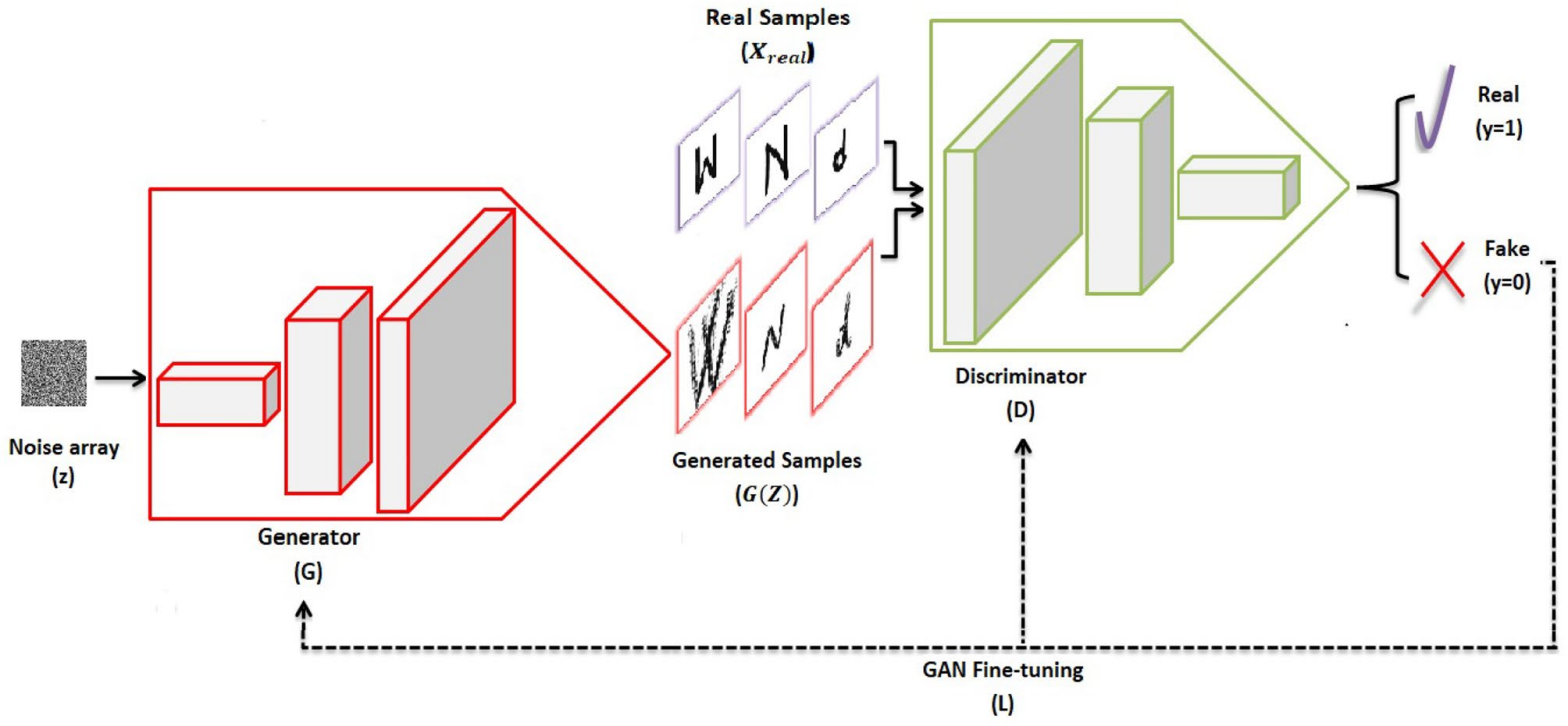
GAN architecture consists of two networks trained simultaneously, *G* and *D*. *G* generates synthetic samples that *D* tries to make plausible.

As presented in Fig. 3, GANs combine two different adversarial networks that are trained simultaneously. The two networks are

1. The generator (*G*): A generative model that is trained to capture the data distribution. It can output synthetic and generative samples from the learned distribution. Random noise (*Z*) is given as an input to *G* for guarantee diversity in the generated output samples.
2. The discriminator (*D*): A discriminative model trained to determine which distribution of the input samples belong. It helps to indicate if they belong to the real data distribution or artificial. *D* works like a teacher who determines whether his student (*G*) needs more practice or if he is working smart. If *D* falls at fault and classifies the artificially generated samples as real, then, it means that the *G* works too well. In summary, *D* improves the total performance in GAN.

During training, the two models compete against each other. *G* tries hard to generate data that appear real and tricks *D*. Simultaneously, *D* also tries not to be deceived and intelligently classifies the input samples. As a result, a zero-sum game is established between the two models, which helps improve their functionalities.

Generally, the *G* function is expressed as $G (z,\theta _ g):Z\rightarrow {\hat{X}}$. It maps the random noise *Z* to the artificial data distribution ${\hat{X}}$ with $\theta _ g$ as the parameters. Furthermore, the *D* function is expressed as $D(x,\theta _ d):(X \cup {\hat{X}}) \rightarrow S$ with$\theta _ d$ as the parameters to differentiate the elements of the real data distribution *X* from ${\hat{X}}$, where *S* is a real number in the interval [0 : 1]. *G* is trained to minimize its loss and maximize *D* loss using Eq. (3)^50^. The objective of *G* is to intelligently generate indistinguishable elements, which *D* classifies as real. *D* is trained simultaneously to minimize its loss using Eq. (4), to not be a cheated and to separate the samples of *X* and ${\hat{X}}$ correctly. In summary, *D* and *G* play a mini-max two-player game and calculate its loss for each single data point using Eq. (5). Thus, the total value function of GAN can be stated as Eq. (6)^24^.

3 $$\begin{aligned} {L^{(G)}}=\min {[}\log D(x) +\log (1-D(G(z))) {]} \end{aligned}$$

4 $$\begin{aligned} {L^{(D)}}=\max {[}\log D(x) +\log (1-D(G(z))) {]} \end{aligned}$$

5 $$\begin{aligned} {L}= \min _{G} \max _{D}{[}\log D(x) +\log (1-D(G(z))) {]} \end{aligned}$$

6 $$\begin{aligned} \min _{G} \max _{D} V(D,G) = {\mathbb {E}}_ {x \sim p_{data} {(x)}} {[}\log D(x) {]} +{\mathbb {E}}_{z\sim p_{z} {(z)}} {[} \log (1-D(G(z))) {]} \end{aligned}$$

### CGANs

There is no control over which classes the generator should produce additional samples in the GAN. It works in an unsupervised mode with no way of requesting particular targeted images. It takes the input as a whole without concentrating on whether the input holds images belonging to different classes or the same class. Then, it starts its role by generating artificial samples that appear real without distinguishing the class to which the artificial sample belongs. Therefore, Mirza and Osindero^24^ proposed CGAN as an extension of the superior performance of GAN from unsupervised learning to supervised learning. CGAN is the supervised version of a GAN in which an extra input layer is added to both the generator and discriminator to guide them in terms of which images should be produced.

**Figure 4:**
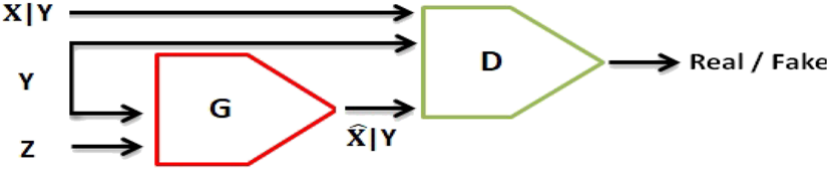
Both *G* and *D* are conditioned with the class labels in CGAN.

CGAN follows the same training style as GAN but with restrictions on the label of the generated samples. As indicated in Fig. 4, *Y* is the additional information that determines the class label for both *G* and *D*. Thus, the cost function for CGAN can be stated as Eq. (7)^24^.

7 $$\begin{aligned} \min _{G} \max _{D} V(D,G) = {\mathbb {E}}_ {x \sim p_{data} (x)} {[} \log D(x|y) {]} +{\mathbb {E}}_ {z\sim p_{z} (z)} {[}\log (1-D(G(z|y))){]} \end{aligned}$$

### Transfer learning specifications

The consideration of transfer learning is stimulated because humans can apply the previously learned knowledge to provide better solutions for new related situations^51^. Transfer learning follows the same style of learning. It creates high-performing learners by extracting knowledge learned from the previously trained models on source tasks. Then, borrow this knowledge to learn new related target tasks. The precise meaning of the borrowed knowledge is the model’s architecture and parameters. Instead, building new deep models from scratch with initialized parameters, transfer learning can be applied. This property aids in making models generalize better and more accessible and tackling the problem of having small data for training newer tasks^52^.

**Figure 5:**
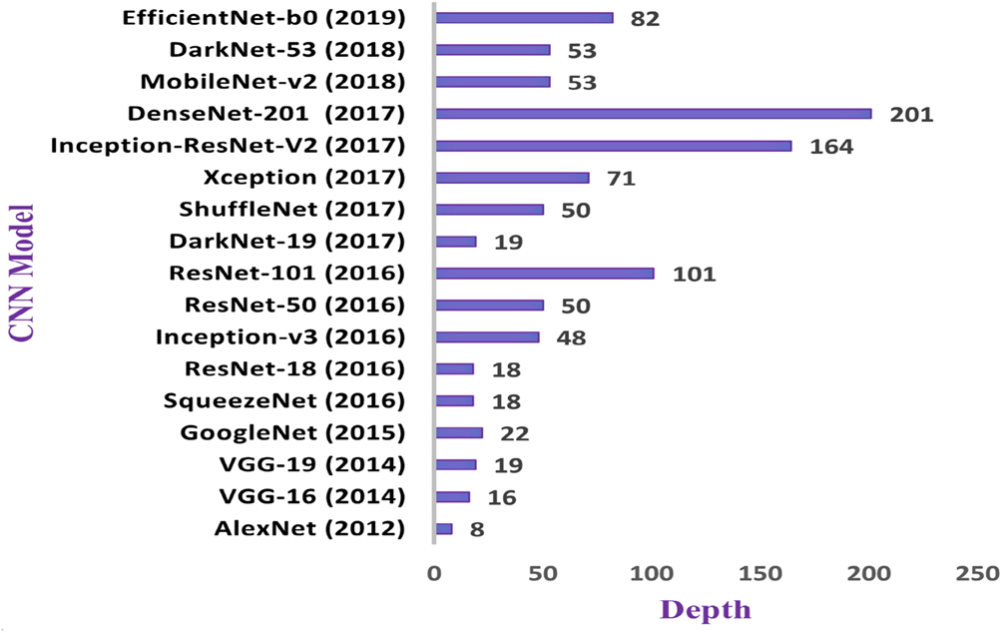
Different transfer learning models with their depth.

Two basic steps are involved when transfer learning is preferred to solve a new target task. The first step is model selection, in which a single model is chosen from the available pretrained ones to be the source model for the new target task. Figure 5 displays the most popular pretrained CNN models that won in the ImageNet Large Scale Visual Recognition Challenge (ILSVRC) from 2012 to 2017^53^. Each model has a different structure and depth and was previously trained on approximately 1.2 million high-resolution images from the ImageNet database to classify 1000 different object categories. Selecting one of these pretrained models for new recognition tasks leads to faster and easier training. While, the second step is model fine-tuning, in which the selected source model is re-designed to suit the new task. Fine-tuning implies that the network is not trained from scratch with initialized weights. Instead, it is trained to adjust its borrowed architecture and parameters to fit the new target task^7,54^. Fine-tuning guarantees that the pretrained model will achieve the best results for the new target tasks. Algorithm 1, presents the basic steps for fine-tuning any pretrained model.

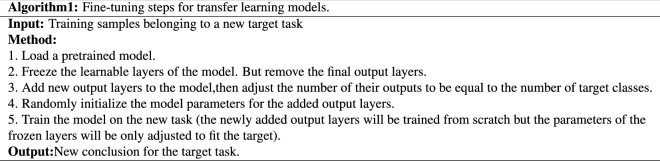



## Using CGAN in fine-tuning transfer learning models for few-Shot HCR tasks

**Figure 6:**
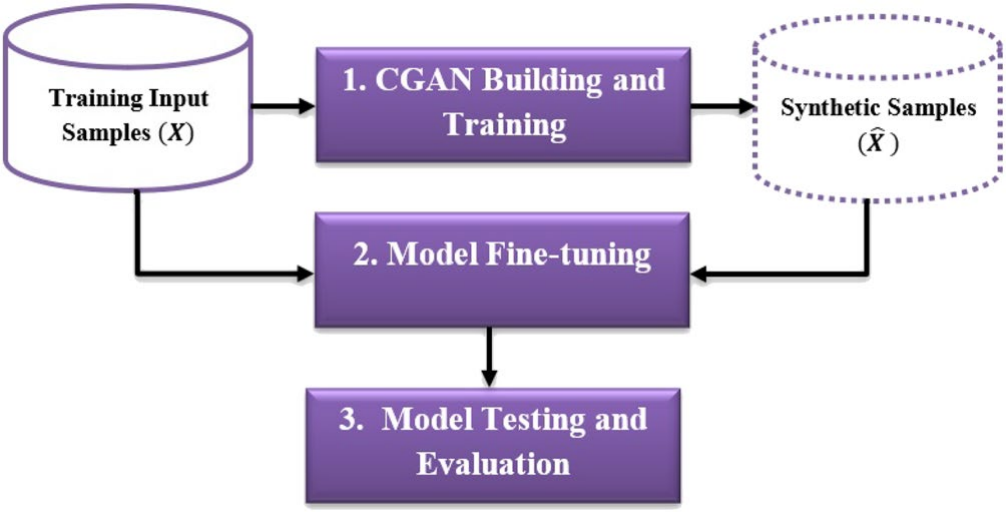
The proposed framework for enhancing the performance of transfer learning models in few-shot HCR tasks.

As mentioned above, applying transfer learning avoids the difficulties of building deep models from scratch. However, fine-tuning transfer learning models in few-shot HCR tasks usually involve an overfitting problem. The pretrained model, during fine-tuning, must see sufficient data variations to acquire generalization ability for handwriting variations. Figure 6 summarizes the proposed framework for solving the overfitting problem, which occurs when applying transfer learning in few-shot HCR tasks. The steps represented in the framework are CGAN building and training, model fine-tuning, and model testing and evaluation. The following subsections present the details of each step.

### CGAN building and training

The purpose of this step is to generate synthetic samples from the few input ones. CGAN is built by two networks that are trained simultaneously, *G* network and *D* network.

#### G network

**Figure 7:**
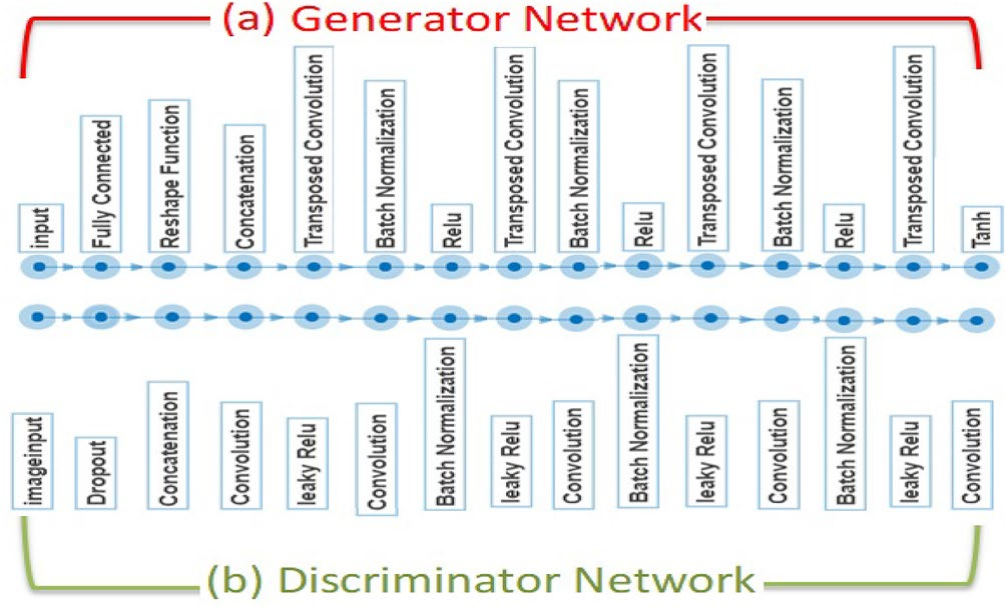
The total structure of G and D networks included in the introduced CGAN architecture.

*G* is built as a series of transposed convolution, batch normalization, and Rectified Linear Unit (RELU) layers. As shown in Fig. 7a, we used 4 transposed convolution, 3 batch normalization, and 3 RELU layers. We set the size of the noise input into *G* to 100 and the embedding dimension for the categorical labels to 50. Similarly, we convert each input using a fully connected layer followed by a reshaping function. The projection size was [ 4 4 1024]. For the transposed convolution layers, $5 \times 5$ filters are applied, with a decreasing number of filters for the following layers. However, in the final transposed convolution layer, three $5 \times 5$ filters are used to represent the three RGB channels of the generated images. Finally, the hyperbolic tangent (*tanh*) activation function is applied to the output layer to produce outputs on the scale of $[-1 ,+1]$.

#### D network

Conversely, *D* is built as a series of convolution, batch normalization, and leaky RELU layers. As presented in Fig. 7b, we used 5 convolutions, 3 batch normalization, and 4 leaky RELU layers. We adjusted the input layer to receive $64 \times 64 \times 1$ images and the corresponding labels. Then, a noise is added to the input by a dropout layer to guarantee the performance of *D*. Similarly, the dropout probability was 0.75. For the convolution layers, $5 \times {5}$filters are used, with an increasing number of filters for the following layers. The scale score of the leaky RELU was set to 0.2.

### Model fine-tuning

The output generated samples by CGAN are used to fine-tune three pretrained models: AlexNet, VGG-16, and GoogleNet. AlexNet is a small-sized network, VGG-16 is a medium-sized network, and GoogleNet is a large-sized network. Choosing different-sized pretrained models shows the extent that the performance of deep learning can be affected by the number of available training samples.

#### AlexNet

AlexNet is a CNN proposed by Krizhevsky et al.^55^. It consists of 25 layers, eight of which are learnable (5 convolutional layers followed by 3 fully connected layers). All learnable layers except the last one use the RELU activation function. The convolutional layers pertain to multiple kernel sizes, which are $11 \times 11$ with 4 strides 0 padding, $5 \times 5$ with 1 stride 2 paddings, and $3 \times 3$ with 1 stride 1 padding. AlexNet performs nonlinear downsampling and reduces the computational complexity by applying three max-pooling functions. Each function uses $3 \times 3$ filters with 2 strides and no padding. For the final fully connected layers, the first two layers have 4096 channels and followed by a dropout layer to prevent overfitting. The last group has 1000 channels, which represent the number of output classes.

#### VGG-16

Zisserman and Simonyan^56^ developed the VGG-16. It comprises 47 layers, 16 of which are learnable (13 convolutional layers followed by 3 fully connected layers). The distribution of the convolutional layers forms five blocks, in which each block ends with a max-pooling layer. Each convolutional layer in each block uses filters with a fixed size, stride, and padding, which are $3\times 3$, 1, and 1, respectively. Additionally, a ReLU activation is performed at the end. VGG-16 uses small and fixed convolutional kernels to reduce the number of used parameters and to conclude more discriminative decision function^57^. The applied max-pooling functions use $2 \times 2$ filters with 2 strides and no padding. Thus, each spatial dimension of the activation map from the previous layer is halved^58^. The final fully connected layers used in VGG-16 are the same as those in AlexNet.

#### GoogleNet

Szegedy et al.^59^ implemented GoogleNet. It consists of 144 layers, 22 of which are learnable. It is also known as Inception-V1 model as it uses the Inception module as its basic unit. The main thought under the Inception module is to run several parallel operations (convolution and pooling) with multiple kernel filter sizes ($1\times 1$, $3 \times 3$, and $5\times 5$) on the same convolutional layer. Then, the output results are concatenated and forwarded to the next convolutional layer. Inception units produce multilevel feature extraction with optimized variants and avoid patch alignment problems^60,61^. They also reduce the number of network parameters. GoogleNet holds seven million parameters which are smaller than the number of parameters in less deep networks such as AlexNet. GoogleNet structure has four convolutional layers, nine Inception modules, four max-pooling layers, three average pooling layers, five fully connected layers, and three SoftMax layers. Additionally, it applies the RELU activation in the convolutional layers and employs dropout regularization in its fully connected layers.

### Model testing and evaluation

Three measures are considered for evaluating each model’s performance. These measures are

1. Validation accuracy (*Val*.*Acc*.): implies the model accuracy on the validation samples and is calculated using Eq. (8).
  8 $$\begin{aligned} {Val.}{Acc.}=\frac{TP + TN}{TP + FN + TN + FB} \end{aligned}$$
  where *TP* is the number of correctly classified samples, *TN* is the number of correctly rejected samples, *FP* is the number of incorrectly rejected samples, and *FN* is the number of incorrectly classified samples.
2. $F1-Score$: covers the harmonic mean of the precision and recall. it assesses summarizing the overall quality of the model and is calculated using Eq. (9)–(11).
  9 $$\begin{aligned} {F1-Score}=\frac{2( Precision \times Recall)}{Precision + Recall} \end{aligned}$$
  10 $$\begin{aligned} {Precision}=\frac{TP}{TP + FB} \end{aligned}$$
  11 $$\begin{aligned} {Recall}=\frac{TP}{TP + FN} \end{aligned}$$
3. Generalization Error $(E_{test})$: represents the model deficiency to recognize new-unseen test samples and is calculated using Eq. (12).
  12 $$\begin{aligned} E_{test}=\frac{1}{n} \sum _{i=1}^{n} {error}{(f_D (x_i), y_i)} \end{aligned}$$
  where *n* is the number of classes, *i* is the number of test samples, $f_{D} (x_{i})$ is the predicted class by the model, and $y_{i}$ is the actual class.

## Experimental results and discussion

Experiments are conducted on a benchmark package of few-shot datasets, which is the Omniglot. All experiments are implemented in MATLAB 2022 *a* on a personal computer Intel Core *i*7 with 2.60 GHz processor and 16*GB* of RAM.

### Datasets description

Omniglot is considered an official package of datasets used to evaluate FSL models^62,63^. It holds 1623 handwritten characters from 50 different languages. Each character was formed by 20 writers and scanned in a grayscale image of size $105 \times 105$. The number of classes in each dataset is equal to the number of language letters the dataset represents. We chose four different languages from Omniglot to work. These languages are Latin, Malay (Jawi-Arabic), Korean, and Sanskrit (old Indo-Aryan). Table 2 summarizes the properties of each dataset. Each dataset is divided into two parts:

- *Part*1: represents $70\%$ of the existing samples used to train the introduced CGAN to generate synthetic samples and as few-shot input to train the transfer learning models.
- *Part*2: represents the rest of the existing samples $(30\%)$ and used as a static test set to perform fair experiments for model evaluation. The network does not see this part during the training. Monitoring the generalization efficiency of the network can be done by measuring the network performance on new-unseen samples.

**Table 2:** Details of the chosen datasets from Omniglot package.

Dataset	No. of classes	No. of samples/class
Latin	26	20
Malay(Jawi-Arabic)	38	20
Korean	40	20
Sanskrit (old Indo-Aryan)	42	20

### CGAN training results

**Figure 8:**
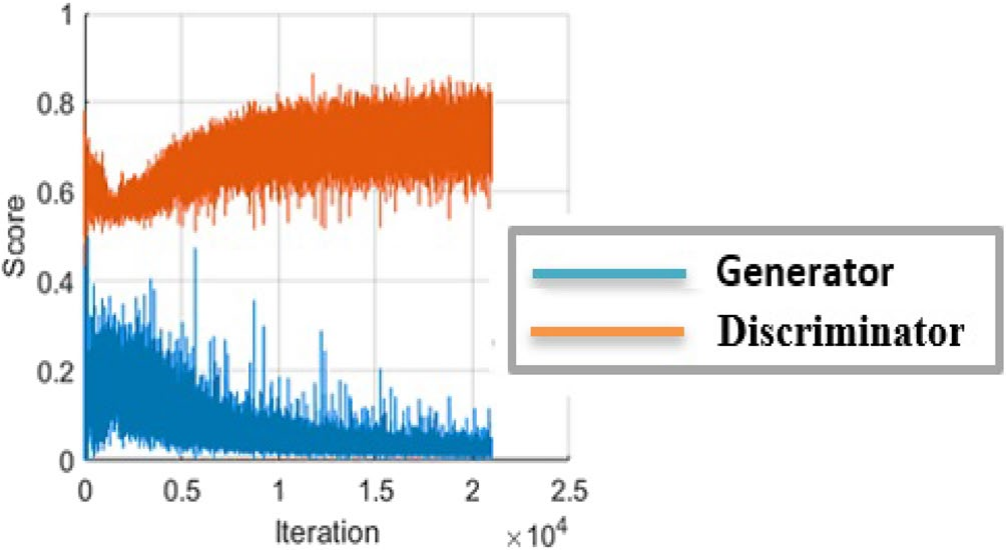
Total loss of the constructed CGAN. *G* loss decreases when the *D* loss increases , the two networks play a mina mini-max two-player game.

**Figure 9:**
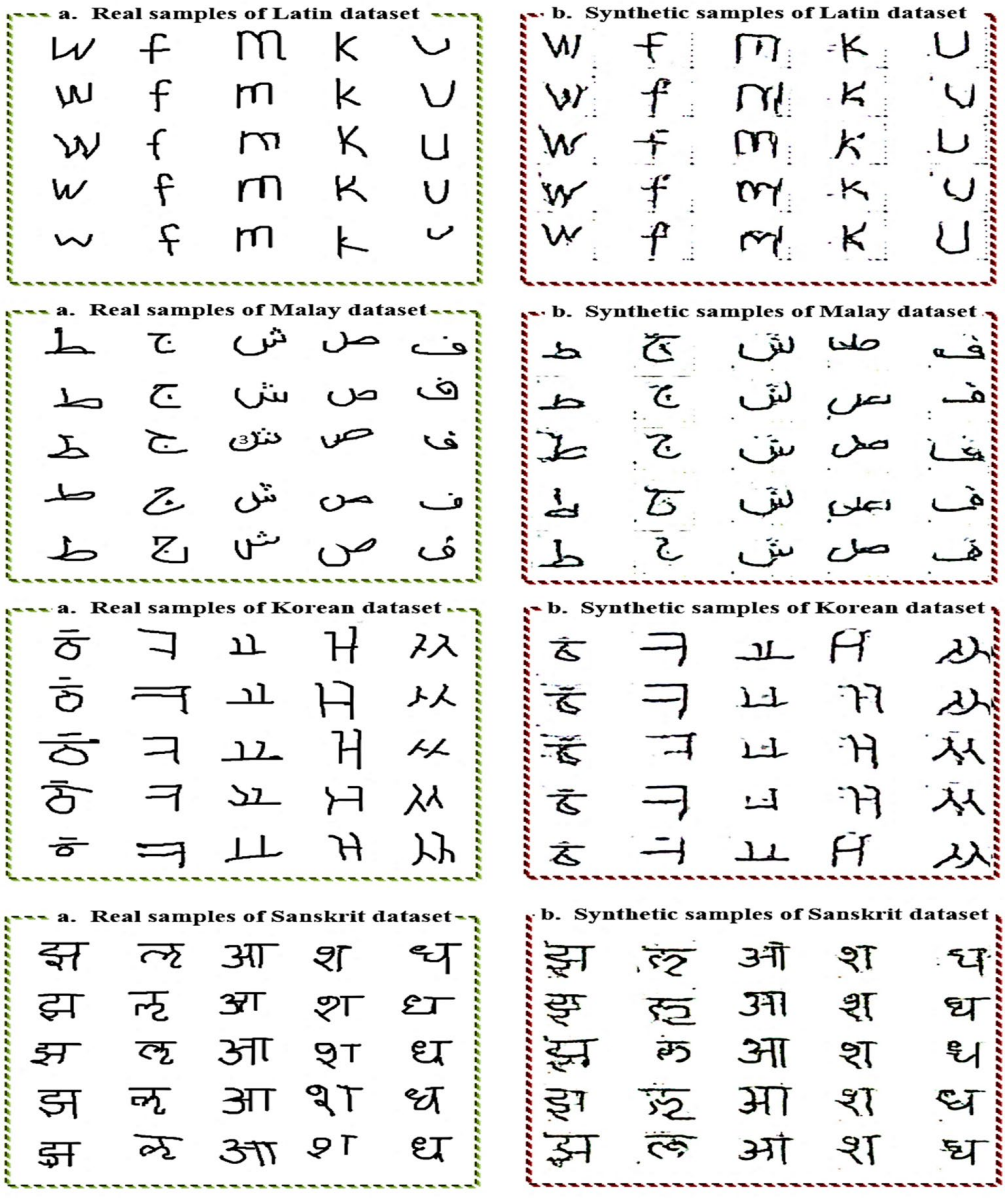
For each dataset: (**a**) represents examples of its real samples, and (**b**) represents examples of its fake samples generated by the construced CGAN.

CGAN was trained for 500 epochs on all datasets. Figure 8 shows its total loss. Notably, *G* loss becomes near or equal to zero in the last iterations, indicating the success of *G* in generating synthetic examples that feel *D* is at fault. Thus, at the *D*, loss becomes large and near 1. Finally, *G* won the min-max two-player game. Figure 9 shows samples of the generated images by GCAN for each dataset.

### Model fine-tuning setting

We edited AlexNet, VGG-16, and GoogleNet to fit the target tasks and placed the final fully connected layer and classification SoftMax layers in all networks with new ones. Similarly, we adjusted the output size of the newly added fully connected layer in each model to equal the number of dataset classes. The input was formed by dividing *part*1 of each dataset into $85\%$ for training and $15\%$ for validation. The applied validation strategy is the holdout cross-validation. Furthermore, we adjusted the learning rate of all networks to 0.0001 and applied the stochastic gradient descent with a momentum optimizer to monitor possible losses. Finally, we trained all the networks for 5 epochs.

### Experimental results

Four training cases are presented in the results. These cases are

1. $Case (A)\rightarrow$ training in the existence of a few samples in each dataset.
2. $Case (B)\rightarrow$ training by applying the traditional image transformations. We performed trial-and-error experiments for each dataset to achieve the optimal transformations that can be used to improve the performance. Table 3 mentions the final transformations that are used in each dataset.
3. $Case (C)\rightarrow$ training with applying the introduced CGAN approach. The number of samples generated by CGAN that are added before training is 500 samples in each class.
4. $Case (A)\rightarrow$ training by applying a combination of the traditional image transformations (*Case*(*B*)) and the introduced CGAN approach (*Case*(*C*)).

**Table 3:** The applied traditional transformations in each dataset for *Case*(*B*).

Dataset	Applied Transformations
Latin	Random reflection with x axis
Malay(Jawi-Arabic)	Random reflection with x-axis, horizontal and vertical translation by a distance in the range $[-3, 3]$ pixels
Korean	Horizontal and vertical translation by a distance in the range $[-3, 3]$ pixels
Sanskrit(old Indo-Aryan)	Random reflection with x-axis, and horizontal translation by a distance in the range $[-5, 5]$ pixels

**Figure 10:**
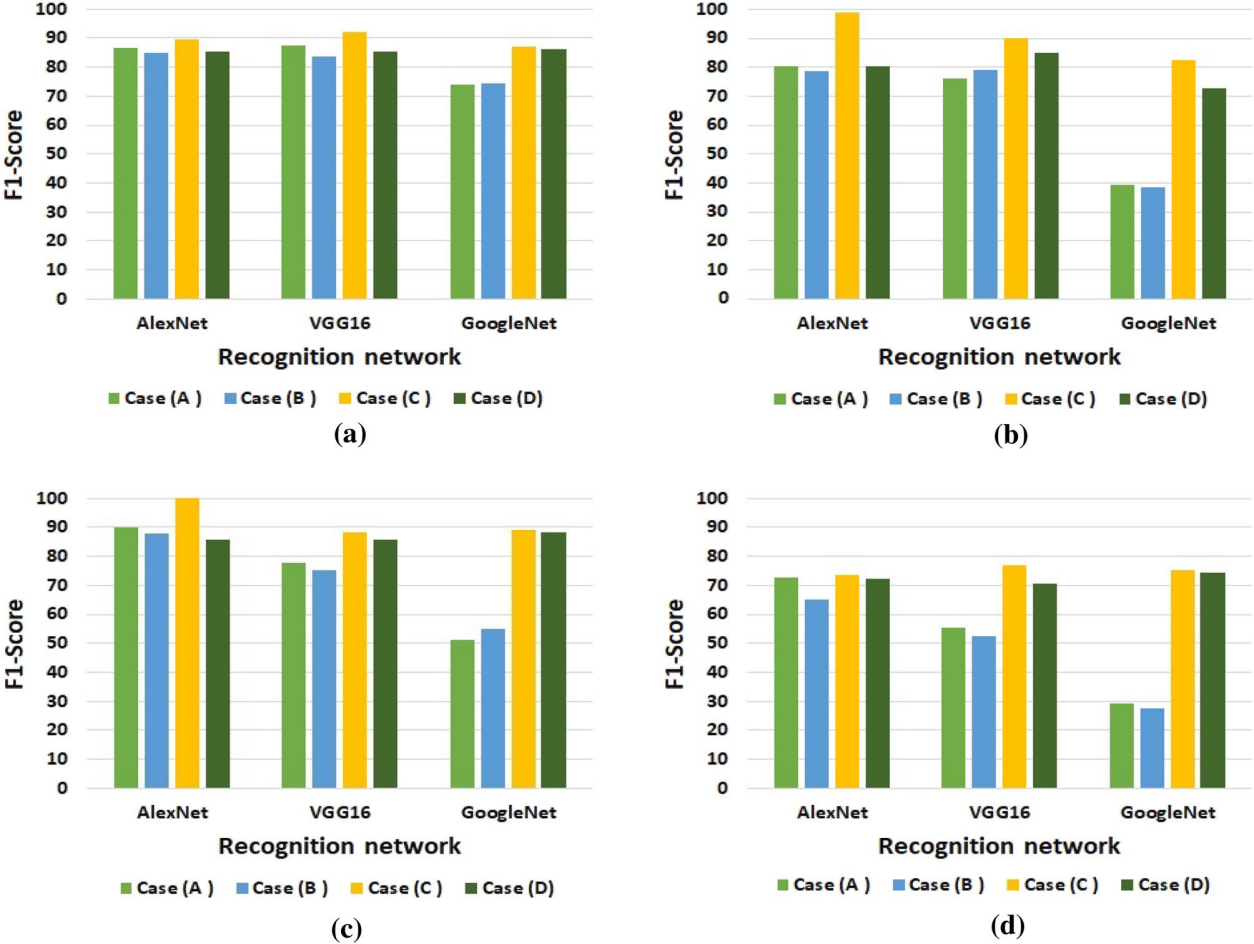
Visualization for the F1-Score recorded by each model under the different training cases for (**a**) Latin dataset, (**b**) Malay(Jawi-Arabic) dataset, (**c**) Korean dataset, and (**d**) Sanskrit(Indo-Aryan) dataset.

Table 4 represents the recognition results. Under the different cases of training, each model records the *Val*.*Acc*., $F1-Score$, and $E_{test}$ measures. Each measure is visualized by a figure to be characterized easily. First, Fig. 10 displays the $F1-Score$ representation for the four datasets. As shown, *case*(*C*) achieves the highest $F1-Score$ and beats all other cases significantly. A high $F1-Score$ indicates the model achieves high value for both Recall and Precision metrics as illustrated in Eq. (9). Formally, comparing among machine learning algorithms is ended when attaining one achieves the highest $F1-Score$. This implies that *case*(*C*) is an optimal training case among all other cases.

**Figure 11:**
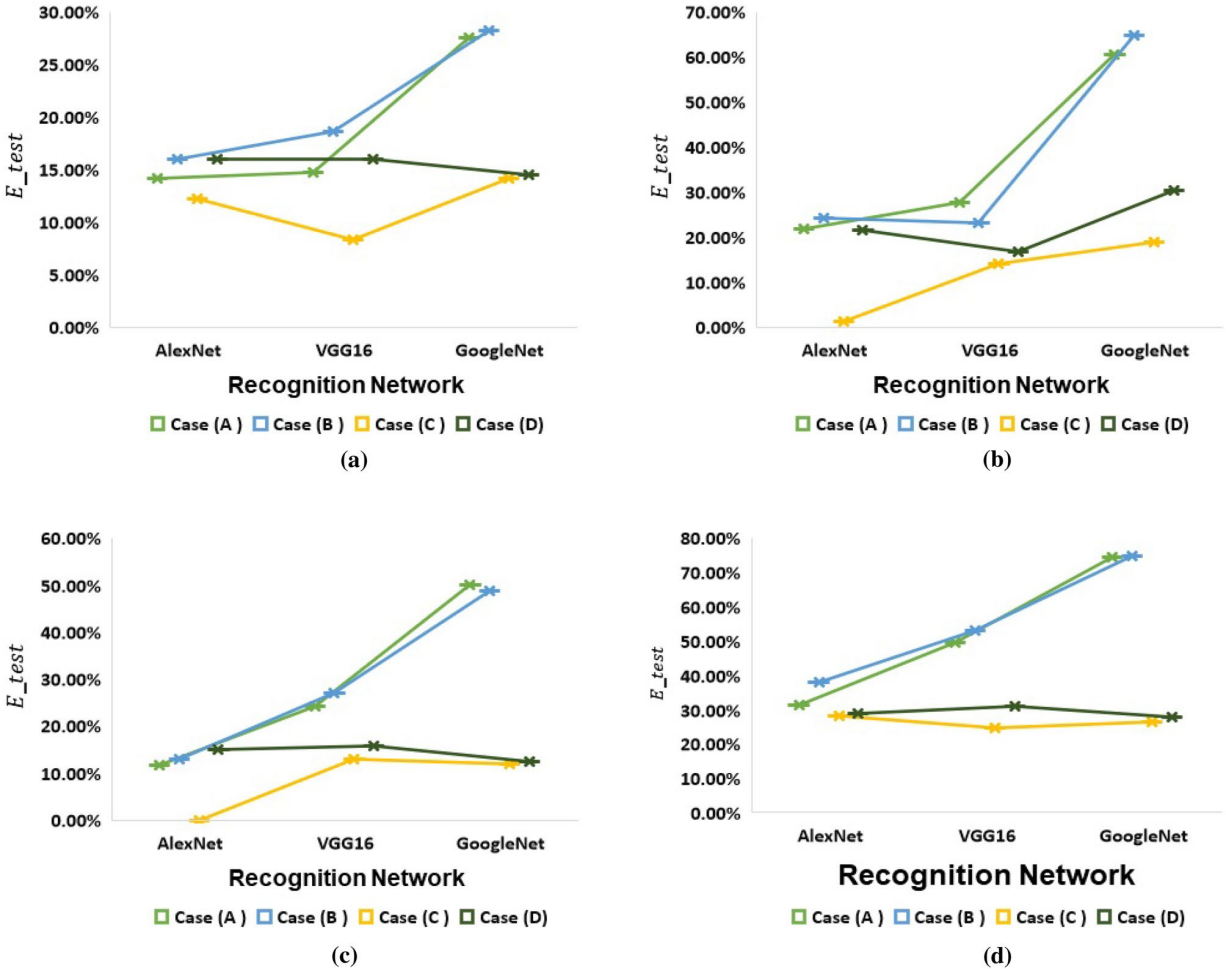
Visualization for the $E_{test}$ recorded by each model under the different training cases for (**a**) Latin dataset, (**b**) Malay dataset, (**c**) Korean dataset, and (**d**) Sanskrit(Indo-Aryan) dataset.

**Figure 12:**
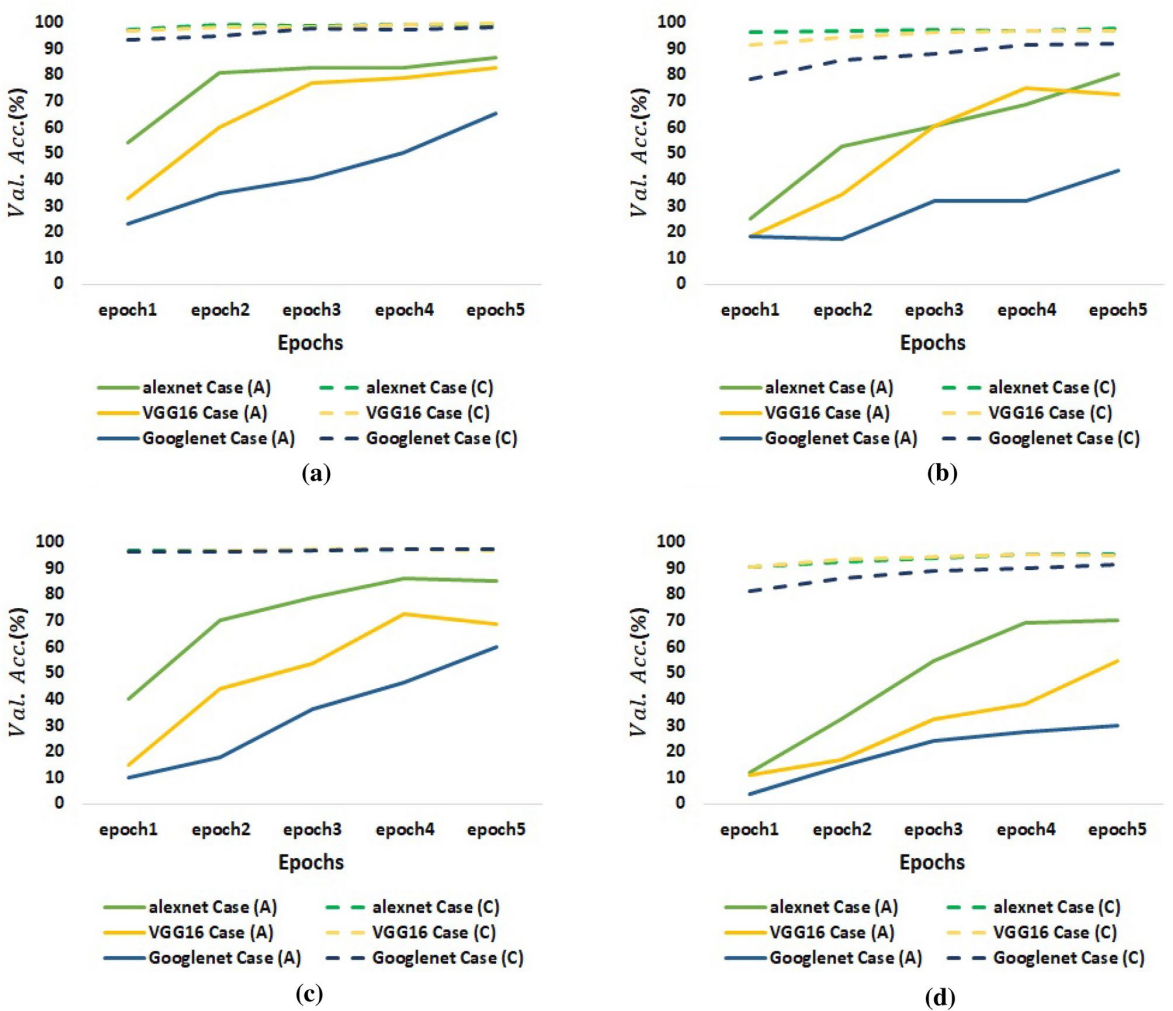
Visualization for *Val*.*Acc*. achieved at each epoch in *Case*(*A*) and *Case*(*C*) for (**a**) Latin dataset, (**b**) Malay dataset, (**c**) Korean dataset, and (**d**) Sanskrit(Indo-Aryan) dataset.

Secondly, Fig. 11 points to the $E_{test}$ occurred by each model in the four datasets. As presented, *case*(*C*) is the case that has the least $E_{test}$ among all cases. Indicating, in *case*(*C*), each model correct recognizes a large number of unseen test samples. Consequently, *case*(*C*) increases the overall generalization ability of the three models. Finally, Fig. 12 displays the recorded *Val*.*Acc*. at each epoch in *case*(*A*)and *case*(*C*). Notably, training with *case*(*C*) achieves high *Val*.*Acc*. at the first two epochs. All models start to converge at the third epoch beginning. We can conclude that *case*(*C*) achieves model fine-tuning in fewer epochs while training with *case*(*A*) needs more epochs.

**Table 4:** The recognition results of AlexNet, VGG-16, and GoogleNet models under the different training cases for each dataset.

Dataset	Recognition Model	$Val. Acc. (\%)$				$F1-Score (\%)$				$E_{test} (\%)$			
		*Case*(*A*)	*Case*(*B*)	*Case*(*C*)	*Case*(*D*)	*Case*(*A*)	*Case*(*B*)	*Case*(*C*)	*Case*(*D*)	*Case*(*A*)	*Case*(*B*)	*Case*(*C*)	*Case*(*D*)
Latin	AlexNet	86.54	80.77	**99.33**	99.19	86.69	85	**89.42**	85.27	14.1	16.03	**12.18**	16.03
	Vgg16	82.69	84.62	**99.36**	98.59	87.24	83.66	**92.20**	85.50	14.74	18.59	**8.33**	16.03
	GoogleNet	65.38	69.23	**98.05**	97.24	73.96	74.29	**86.98**	86.31	27.56	28.21	**14.1**	14.47
Malay(Jawi-Arabic)	AlexNet	80.26	89.47	**97.57**	92.36	80.18	78.55	**98.82**	80.12	21.93	24.12	**1.32**	21.49
	Vgg16	72.37	76.32	**96.79**	92.61	76.18	79.08	**89.93**	84.85	27.63	23.25	**14.04**	16.76
	GoogleNet	43.42	47.37	**91.78**	86.89	39.46	38.4	**82.60**	72.52	60.53	64.91	**18.86**	30.26
Korean	AlexNet	85	90	**97.24**	96.80	89.83	88.05	**100**	85.80	11.76	12.92	**0**	15
	Vgg16	68.75	72.50	**96.67**	97.27	77.81	75.36	**88.12**	85.77	24.12	27.08	**12.92**	15.83
	GoogleNet	60	47.50	**96.98**	96.82	51.34	55.02	**89.10**	88.45	50	48.75	**12.08**	12.50
Sanskrit(old Indo-Aryan)	AlexNet	70.24	78.57	**95.21**	93.12	72.47	65.28	**73.47**	72.42	31.35	38.10	**28.17**	28.97
	Vgg16	54.76	61.90	**94.82**	91.56	55.54	52.51	**77.01**	70.43	49.60	53.17	**24.60**	30.95
	GoogleNet	29.76	30.95	**91.50**	87.73	29.24	27.37	**75.11**	74.45	74.60	75	**26.59**	27.78

Significant values are in [bold].

### Discussion

We used four datasets for different languages to evaluate the proposed framework for solving the overfitting problem, which occurs when applying transfer learning in few-shot HCR tasks. These languages are Latin, Malay (Jawi-Arabic), Korean, and Sanskrit (old Indo-Aryan). Each dataset had only 20 labeled handwritten samples in each class. We divided these labeled samples into $70\%$ for training and $30\%$ for testing. The training set in each dataset is used to train a CGAN for generating the synthetic samples and to train the transfer learning model. We executed various training cases to compare and evaluate the proposed framework. These cases are *A* (training with the few existing input samples), *B* (training by applying the traditional transformations to the few input samples), *C* (training by adding the samples generated by the CGAN to the few input samples, and *D* (training by combining case *B* and case *C*). We used the test set to evaluate each model’s performance under each training case. The transfer learning models which are trained in the experiments are the AlexNet, VGG-16, and GoogleNet. These models differ in their deep structure. AlexNet is the smallest network, whereas GoogleNet is the deepest network. We can conclude with the following highlights from all conducted experiments:

- For *case*(*A*): the $F1-Score$ and $E_{test}$ recorded by each model are significantly different. AlexNet records the highest $F1-Score$ and the least $E_{test}$ in all datasets. GoogleNet records the least $F1-Score$ And the highest $E_{test}$. while VGG-16 achieves records in between. This reveals that the depth of the network is an important factor that controls the performance of transfer learning models in few-shot HCR tasks. Deeper networks achieve higher $E_{test}$ if they are trained with small number of training samples. For the *Val*.*Acc*. , the records of the three models at the first epochs are not high. The *Val*.*Acc*. of AlexNet, VGG-16, and GoogleNet at the third epoch in the Sanskrit dataset reaches $54.76\%$, $32.14\%$, and $23.8\%$ respectively. Even though, applying transfer learning makes the model need fewer epochs for training. But the few existing input samples cause very high loss for the model at the first epochs.
- For *case*(*B*): the advantage of applying the traditional transformations appears in the performance of two models in two different datasets, VGG-16 for Malay dataset and GoogleNet for Korean dataset. In these two situations, *case*(*B*) achieves higher $F1-Score$ and lower $E_{test}$ than *case*(*A*). However, in the rest, *case*(*B*) achieve the worst results among all cases. Results prove that applying the traditional transformations is not suitable as a generalized data augmentation strategy for all few-shot HCR datasets.
- For *case*(*C*): applying the introduced CGAN approach achieves the highest $F1-Score$ and the least $E_{test}$ in all models and for all datasets. The $E_{test}$ for AlexNet in Korean dataset reaches $0\%$ while it is $11.76\%$ , $12.92\%$, and$15\%$ in *case*(*A*), (*B*), *and*(*D*) respectively. Also, the $F1-Score$ for GoogleNet in Sanskrit dataset reaches $75.11\%$ while it is $29.24\%$, $27.37\%$,and $74.45\%$ in *case*(*A*), (*B*), and (*C*) respectively. Additionally, each model in *case*(*C*) reaches to fast convergence. As shown in Fig. 12, at the beginning of the third epoch , all models reach the highest *Val*.*Acc*. and start to converge. So, results prove that using the introduced CGAN approach is an effective solution for reducing the overfitting problem when applying transfer learning in few-shot HCR tasks.
- For *case*(*D*): Combining traditional transformations with CGAN increases the $F1-Score$ in all models compared with *case*(*A*), *and*(*B*). However, its effect in the $E_{test}$ differs. It works better and achieves lower $E_{test}$ than *case*(*A*)*and*(*B*) in GoogleNet and VGG-16 for all datasets. But it achieves higher $E_{test}$ than *case*(*A*), *and*(*B*) in AlexNet for Latin and Korean dataset. The usefulness of *case*(*D*) may appear in deeper networks. But comparing with *case*(*C*), *case*(*D*)achieves lower F1-Score and higher $E_{test}$ than *case*(*C*) in all models and for all datasets. Results prove that *case*(*C*) is more effective and generalized than *case*(*D*).

In summary, results prove that adding synthetic samples generated by CGAN in training transfer learning models for few-shot HCR tasks helps in increasing the total performance of the model, avoiding overfitting, and achieving model fine-tuning in fewer epochs. However, the implemented CGAN in our study is the vanilla CGAN. Improving CGAN implementation by several optimizations such as enhancing the topology of G and D networks, preprocessing inputs before passing them to CGAN, and improving the loss function may allow an additional great result.

## Conclusion and future work

We introduced a proposed framework for solving the overfitting problem which occurs when applying transfer learning in few-shot HCR tasks. The proposed framework considers using a CGAN approach to enlarge the number of the few existing input training samples. CGAN is an improvement for the general GAN and helps in preserving stable and faster training and generating better-quality artificial data. We evaluated the proposed framework in training AlexNet, VGG-16, and GoogleNet models for four few-shot HCR datasets from the Omniglot package. These datasets are Latin, Malay (Jawi-Arabic), Korean, and Sanskrit (old Indo-Aryan). Results show that training with the addition of the generated synthetic samples by CGAN reaches fast convergence in few epochs and achieves the highest *Val*.*Acc*., $F1-Score$, and the least $E_{test}$ measures compared with three other training cases. These cases are training with the few existing input samples, training by applying the traditional transformations to the few input samples, and training with combining the traditional transformations with CGAN.

For future work, we aim to test the performance of pretrained Vision Transformer (ViT) models in few-shot HCR tasks. The Transformer architectures have become the top standard for Natural Language Processing (NLP); however, their use cases in computer vision tasks are limited. So, the performance of ViT models in few-shot image classification tasks needs exploring.

## Data Availability

The datasets generated and/or analysed during the current study are available in the **[github]** repository,at https://github.com/brendenlake/omniglot.
